# mir-21 Overexpressing Mesenchymal Stem Cells Accelerate Fracture Healing in a Rat Closed Femur Fracture Model

**DOI:** 10.1155/2015/412327

**Published:** 2015-03-23

**Authors:** Yuxin Sun, Liangliang Xu, Shuo Huang, Yonghui Hou, Yang Liu, Kai-Ming Chan, Xiao-Hua Pan, Gang Li

**Affiliations:** ^1^Department of Orthopaedics & Traumatology, Faculty of Medicine, The Chinese University of Hong Kong, Prince of Wales Hospital, Shatin, Hong Kong; ^2^Stem Cells and Regenerative Medicine Laboratory, Li Ka Shing Institute of Health Sciences, The Chinese University of Hong Kong, Prince of Wales Hospital, Shatin, Hong Kong; ^3^The CUHK-ACC Space Medicine Centre, The Chinese University of Hong Kong, Shenzhen Research Institute, Shenzhen 518000, China; ^4^Department of Orthopaedic Surgery, Baoan Hospital of Southern Medical University, The 8th People's Hospital, Shenzhen 518000, China; ^5^MOE Key Laboratory for Regenerative Medicine, School of Biomedical Sciences, Faculty of Medicine, The Chinese University of Hong Kong, Shatin, Hong Kong

## Abstract

MicroRNAs are small noncoding RNAs involved in numerous biological processes. Emerging pieces of evidence suggest that microRNAs play important roles in osteogenesis and skeletal homeostasis. Recent studies indicated the significant regulation function of mir-21 in osteogenesis *in vitro*, but little information is known about its veritable functions *in vivo*. In the present study, we aimed to investigate the effect of mir-21 intervention on osteogenic differentiation of rats bone marrow derived mesenchymal stem cells (rBMSCs) and repair capacity in rats closed femur fracture model with internal fixation. The results showed that the upregulation of mir-21 not only increased the expression of osteopontin and alkaline phosphatase in rBMSCs but also promoted mineralization in the condition of osteogenic induction. Furthermore, the bone healing properties were also improved in fracture healing model according to the results of micro-CT, mechanical test, and histological analysis. The current study confirms that the overexpression of mir-21 could promote osteogenesis and accelerate bone fracture healing, which may contribute to a new therapeutic way for fracture repair.

## 1. Introduction

Fractures are the most common large-organ, traumatic injuries in humans [1]. Although the bone has the ability of self-renewal, there are still approximately 10–20% of fractures resulting in impaired or delayed healing [2]. Therefore, there is a burning need to develop therapeutic strategies to accelerate bone regeneration in fracture healing process.

Fracture healing is a complex biological process involving four stages: inflammatory response, soft callus formation, hard callus formation, and bone remodeling [3]. The regeneration process of fracture healing is precisely organized by hormones, cytokines, chemokines, growth factors, and other regulators in each stage.

Mesenchymal stem cells (MSCs) have multipotent capacity to differentiate into a variety of cell types, including osteoblasts, adipocytes, chondrocytes, myoblasts, and neurons [4, 5]. MSCs have been reported to be involved in several stages of fracture healing process. Initially, MSCs could migrate into the fracture site from blood, periosteum, bone marrow, and other tissues. Then osteoblasts and chondrocytes are formed due to the proliferating and differentiating of MSCs. Consequently, immature bone is developed by calcification of osteoblasts and chondrocytes. Finally, bone remodeling is activated together with osteoclasts. Based on these understandings, exogenous intervention of MSCs in fracture healing has been reported to be feasible and available. Besides, it has been demonstrated that MSCs derived from bone marrow (BMSCs) rather than those derived from adipose showed more potential for osteogenic differentiation [6], which is currently the most common source of osteogenous seed cells in bone regeneration.

The regeneration process of fracture healing involving stem cells is precisely organized by hormones, cytokines, chemokines, growth factors, and other regulators in each stage. Recently studies demonstrated that microRNAs are also involved in the process of fracture healing [7, 8].

MicroRNAs are noncoding small RNAs, 21–25 nt in length, encoded in the genome, which can regulate the gene expression by targeting 3′-untranslated region (UTR) of mRNAs at posttranscriptional level. Emergency evidence indicates that miRNAs play important roles in skeletal development and homeostasis [9–11]. Therefore the signatures of these miRNAs could reflect associations with skeletal disorders, such as osteoporosis, osteoarthritis, and osteosarcoma. For example, mir-503 was found markedly reduced while mir-133a was found significantly upregulated in peripheral blood mononuclear cells of postmenopausal osteoporosis patients, respectively [12, 13]. Jones et al. found that mir-9 and mir-98 were identified to be overexpressed in human osteoarthritic tissue [14]. Lulla et al. identified that mir-135b, mir-150, mir-542-5p, and mir-652 were differentially expressed in osteosarcoma compared with normal osteoblasts [15]. As a consequence, the pattern change of miRNAs could be the potential targets for clinical intervention and numerous studies characterizing miRNAs function in relation to their targets during osteoblast or osteoclast differentiation [16, 17]. These bone-regulating miRNAs can even be described as “osteomiRs” [18, 19]. One of the most studied microRNAs, mir-21, is recognized as a versatile miRNA which is involved in lots of biological processes, including osteogenesis [20]. mir-21 was found highly expressed during osteogenic differentiation, which indicated that mir-21 may possibly repress stemness maintenance in osteoblasts [19]. Besides, upregulation of mir-21 by using mir-21 precursor was demonstrated to be beneficial to osteogenic differentiation of MSCs* in vitro* and also promoted ectopic bone formation* in vivo* [21]. However, opposite research results pointed that overexpression of mir-21 was related to the osteoclastogenesis [22], adipogenesis [23], and osteolysis [24]. Mineralization was also found to be suppressed as a result of the upregulation of mir-21 [25]. The conflict results illustrated that the regulation of mir-21 was complicated. Therefore, the osteogenic function of mir-21 in regeneration should be researched intensively and comprehensively. Moreover, most of our knowledge about the function of mir-21 in osteogenesis still stays at the level of* in vitro* study. The understanding about mir-21 functions in fracture healing* in vivo* remains to be unknown. Considering that the microenvironment of fracture site is more sophisticated than that of* in vitro*, the osteogenic regulation of mir-21 needs to be confirmed before it could be applied in clinical setting.

## 2. Materials and Methods

### 2.1. Animals

Four-week-old and 12-week-old SD male rats were obtained from the Laboratory Animal Research Centre of The Chinese University of Hong Kong. Bone marrow-derived mesenchymal stem cells (rBMSCs) of 4-week-old rats were used for culture. 12-week-old rats were used for standard closed transverse femoral fracture model with internal fixation. All animal experimental research protocols were reviewed and approved by Animal Experimentation Ethics Committee of The Chinese University of Hong Kong.

### 2.2. Culture of rBMSCs

The rBMSCs were isolated as previously described [26]. Briefly, the rBMSCs were obtained from the bone marrow of 4-week-old SD rat and cultured in a 100 mm cell culture dish in the alpha complete culture medium at 37°C with 5% CO_2_ and 95% humidity. The rBMSCs from passages 3–8 were used in the experiments. The surface antigens of rBMSCs were detected by flow cytometry using CD90, CD44, CD34, and CD45 (data not shown).

### 2.3. Overexpression of mir-21 in rBMSCs

To generate pLL3.7-pre-mir-21, the oligonucleotides encoding pre-mir-21 were amplified and cloned into the XhoI site of pLL3.7 under the control of U6 promoter. Scrambled control plasmid was also constructed according to the method used by Splinter et al. [27]. The pseudolentiviruses were produced by transfection of 293FT packaging cells (Invitrogen, USA) using the calcium phosphate method. For transduction, 1 × 10^5^ cells were seeded into 6-well plate and incubated with lentiviruses and 8 *μ*g/mL polybrene in the incubator for 24 h [28].

### 2.4. Osteogenic Differentiation and Alizarin Red S Staining

24 hours after the transfection, osteogenic differentiation was performed as previously described [29]. Briefly, the medium was removed and replaced by osteogenic induction medium (1 nM dexamethasone, 50 mM L-ascorbic acid-2-phosphate, and 20 mM *β*-glycerophosphate with complete medium). The induction medium was changed every 3 days. Total RNA was extracted at 3 and 7 days after induction for quantitative real time PCR analysis. 14 days after the induction, Alizarin Red S staining was performed to evaluate calcium deposits formation. To quantify the staining, cultures were destained using 10% cetylpyridinium chloride (CPC) in 10 mM sodium phosphate, pH 7.0, for 15 min at room temperature. Aliquots of exacts were diluted 10-fold in 10% CPC solution, and Alizarin Red S concentration was determined by absorbance measurement at 550 nM on a multiplate reader (Thermo-Labsystems, Leuven, Belgium).

### 2.5. RNA Extraction and Quantitative Real-Time PCR

Total cellular RNA was isolated with RNA Mini Kit (Invitrogen) and then reverse-transcribed into cDNA using M-MLV Reverse Transcriptase (Invitrogen) according to the manufacturer's instructions. Real-time PCR was performed using the Step One Plus Real-Time PCR System (Applied Biosystems, USA) according to the manufacturer's instructions. The reaction conditions consisted of 15 *μ*L reaction volumes with diluted cDNA template 3 *μ*L, 7.5 *μ*L SYBR-Green Master Mix (2×), 3.9 *μ*L PCR-grade water, and 0.3 *μ*L of each primer (10 *μ*M). Amplification conditions were as follows: first at 95°C for 5 min and then 40 cycles of 95°C for 15 s and 60°C for 60 s. Primer sequences were listed in Supplementary Table  1 in Supplementary Material available online at http://dx.doi.org/10.1155/2015/412327. The relative quantification of gene expression was analyzed with 2^−ΔΔCT^ method, normalized with *β*-actin expression level.

### 2.6. Quantitative RT-PCR for mir-21

Total RNA was extracted with TRIzol (Invitrogen) and then reverse-transcribed into cDNA using M-MLV Reverse Transcriptase (Invitrogen) and miRNAs were collected with All-in-One miRNA quantitative reverse transcription- (qRT-) PCR detection kit (GeneCopoeia, Guangzhou, China) according to the manufacturer's instructions. Real-time PCR was performed using the Step One Plus Real-Time PCR System (Applied Biosystems, USA), as indicated in the instructions. To analyze the expression levels of the mRNAs, the reaction conditions consisted of 15 *μ*L reaction volumes with diluted cDNA template 3 *μ*L, 7.5 *μ*L SYBR-Green Master Mix (2×), 3.9 *μ*L PCR-grade water, and 0.3 *μ*L of each primer (10 *μ*M). Amplification conditions were as follows: first at 95°C for 5 min and then 40 cycles of 95°C for 15 s and 60°C for 60 s. To analyze the expression level of mir-21, a total reaction volume of 20 *μ*L contained 10 *μ*L SYBR Mix, 5.6 *μ*L RNase-free water, 1 *μ*L mir-21 primer, 1 *μ*L universal adaptor PCR primer, 2 *μ*L cDNA template, and 0.4 *μ*L ROX. Amplification and detection were performed as follows: 95°C for 10 min and then 40 cycles of 95°C for 15 s, 60°C for 30 s, and 72°C for 20 s. Primer sequences were listed in Supplementary Table  1. The relative quantification of gene expression was analyzed with 2^−ΔΔCT^ method, normalized with *β*-actin expression level.

### 2.7. Animal Surgery

Standard rat closed transverse femoral fracture model with internal fixation was used in this study. Briefly, eighteen 12-week-old SD male rats were under general anaesthesia and sterile condition, a small incision was made at medial knee, and a hole was drilled at the intercondylar notch with an 18-gauge needle (Terumo). A K-wire (diameter: 1.2 mm, Stryker Ltd, USA) was inserted into the right femoral bone marrow cavity. After incision was sutured, a closed fracture was produced at the midshaft of the right femur using a custom-made 3-point bending device, with a metal dull blade (weighted 500 g) dropping from a height of 35 cm, and X-ray was taken to confirm the fracture. All the animals were randomly and equally assigned into 2 groups after the surgery: mir-21 treatment groups and control groups.

### 2.8. Local Injection of rBMSCs

The rBMSCs stably overexpressed mir-21 and scramble control were harvested with 2.5% trypsin and resuspended into the PBS. A total of 1 × 10^6^ cells were prepared for each animal. The cells were locally injected into the fracture site of the bone under the monitoring of the X-rays 7 days after the surgery.

### 2.9. Microcomputer Tomography (Micro-CT) Examination

All animals were sacrificed 5 weeks after fracture and micro-CT analysis was performed for each animal as previously described [30]. Briefly, all the specimens were imaged using a vivaCT 40 (Scanco Medical) with a voltage of 70 keV, a current of 114 *μ*A, and 10.5 *μ*m isotropic resolution. The fracture site was selected as the volume of interest. Low- and high-density mineralized tissues were reconstructed using different thresholds (low attenuation = 160, high attenuation = 350) using our established evaluation protocol with small modification [31]. The high-density tissues represented the newly formed highly mineralized calluses and the old cortices, while the low-density tissues represented the newly formed calluses. Bone volume (BV), tissue volume (TV), and BV/TV of each sample were recorded for analysis.

### 2.10. Four-Point Bending Mechanical Testing

Mechanical test was performed within 24 hours after sacrifice at room temperature; the contralateral femora were also tested as an internal control. A four-point bending device (H25KS; Hounsfield Test Equipment Ltd. UK) with a 50 N load cell was used to test the femur to failure. The femurs were loaded in the anterior-posterior direction with the inner and outer span of the blades set as 8 and 20 mm, respectively. The long axis of the femora was oriented perpendicular to the blades during the test (19). The ultimate load (UL), the energy to failure, and the modulus of elasticity (*E*-modulus) were recorded and analyzed using built-in software (QMAT Professional; Tinius Olsen, Inc., Horsham, PA, USA). The biomechanical properties of the healing fractures were expressed as percentages of the contralateral intact bone properties.

### 2.11. Histological Analysis

The femora were fixed in 10% buffered formalin, decalcified with 9% formic acid, and embedded in paraffin. Attempts were made to standardize the sectioning at a midsagittal plane of each specimen by cutting the specimen in half (longitudinally in a sagittal plane) using a slicing blade. Thin sections (5 *μ*m) are cut by a Rotary Microtome (HM 355S, Thermo Fisher Scientific, Inc., Germany) along the long axis of each femur in sagittal plane. Hematoxylin and eosin (HE) and Safranin O staining were performed using standard protocols after deparaffinization.

### 2.12. Statistical Analysis

All quantitative data were transferred to statistical spreadsheets and analyzed by a commercially available statistical program SPSS version 16.0 (IBM, USA); independent *t*-test was used for comparison of mean values with *P* < 0.05 considered as statistically significant.

## 3. Result

### 3.1. mir-21 Promoted Mineralization of rBMSCs

**Figure 1:**
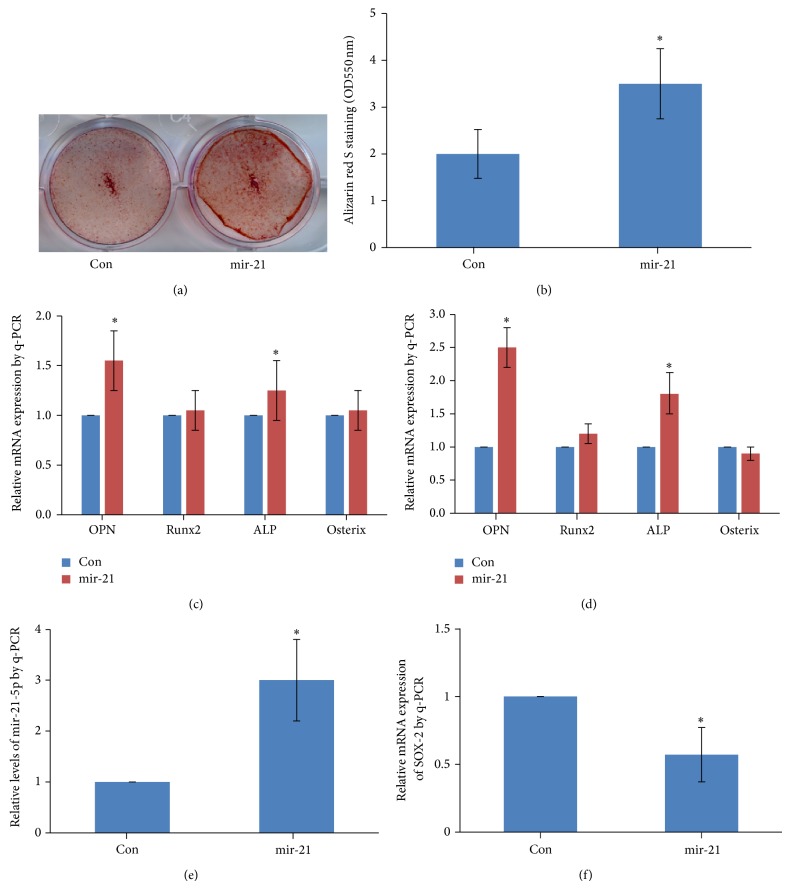
mir-21 promoted osteogenesis of rBMSCs* in vitro*. (a) rBMSCs were treated with mir-21 in osteogenic induction medium for 14 days. Mineralized nodules were stained by Alizarin Red S. (b) Alizarin red S concentrations were quantified by absorbance measurement at 550 nM. (c-d) rBMSCs were treated with mir-21 in osteogenic induction medium for 3 (c) and 7 days (d). Osteogenesis-related gene expressions were detected by q-PCR. (e) mir-21-5p expression level was detected 3 days after osteogenic induction. (f) SOX-2, the potential target of mir-21, was also detected 3 days after osteogenic induction. *β*-actin was used as internal control. The experiments were repeated three times. ^*^ *P* < 0.05, compared with control.

To testify the effect of mir-21 on osteogenesis of rBMSCs* in vitro*, Alizarin Red S staining was performed at day 14 after mir-21 transfection (Figure 1(a)). The quantitative result of Alizarin Red showed that overexpression of mir-21 could remarkably increase calcium nodule formation compared with the control group (Figure 1(b)). Moreover, we detected the gene expression of OPN, Runx2, ALP, and Osterix which are the markers of osteoblastic differentiation. The q-PCR result showed that OPN and ALP were both significantly upregulated by mir-21 at day 3 and day 7 in presence of osteogenic induction medium (Figures 1(c) and 1(d)). As the level of mir-21-5p was more abundant than mir-21-3p, so we detected the level of mir-21-5p by q-PCR after lentivirus infection. The result showed that mir-21-5p was upregulated about 3 times (Figure 1(d)). Besides, the expression level of mir-21 and its potential target SOX-2 were also detected 3 days after osteogenic induction; the results showed that SOX-2 decreased as the increase of mir-21 abundance, indicating that there was a negative correlation between mir-21 and SOX-2 (Figure 1(e)).

### 3.2. Radiographic Analysis of Fractured Femur

**Figure 2:**
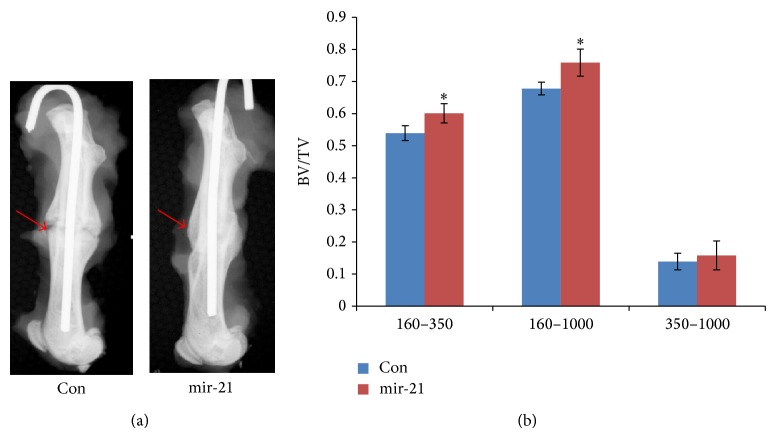
mir-21-MSCs promoted fracture healing* in vivo* via radiologic analysis. (a) X-ray was taken 5 weeks after fracture with a high-resolution digital radiograph system (Faxitron MX-20, Illinois, USA) using an exposure of 32 kV for 10 seconds. (b) Micro-CT analysis data showed that BV/TV of newly formed bone in mir-21-MSCs group was much higher than that in control which prompted that the elevation of mir-21 accelerated the deposition of newly formed bone. Attenuation above 160 represented total mineralized tissue, and attenuation between 160 and 350 represented the newly formed calluses. ^*^ *P* < 0.05, compared with control.

In order to determine whether mir-21 transfected rBMSCs could accelerate bone fracture healing, closed femur fractures were created in 8-week-old SD rats. mir-21-MSCs or Con-MSCs were injected locally at 4 days after fracture. At 5 weeks after fracture, the X-ray showed that the gap in the fracture site disappeared in mir-21-MSCs treated group (Figure 2(a)). Conversely, an obvious gap was found at fracture sites in control group (Figure 2(a)). Besides, the callus width of fractured femurs was much smaller in the mir-21 treatment group than that of control group, meaning that bone remodeling was partially accomplished in mir-21 treatment group. The value of BV/TV calculated by micro-CT indicated that much more newly formed mineralized bone could be detected in mir-21 group compared to the control group 5 weeks after fracture (Figure 2(b)).

### 3.3. Histological Analysis

**Figure 3:**
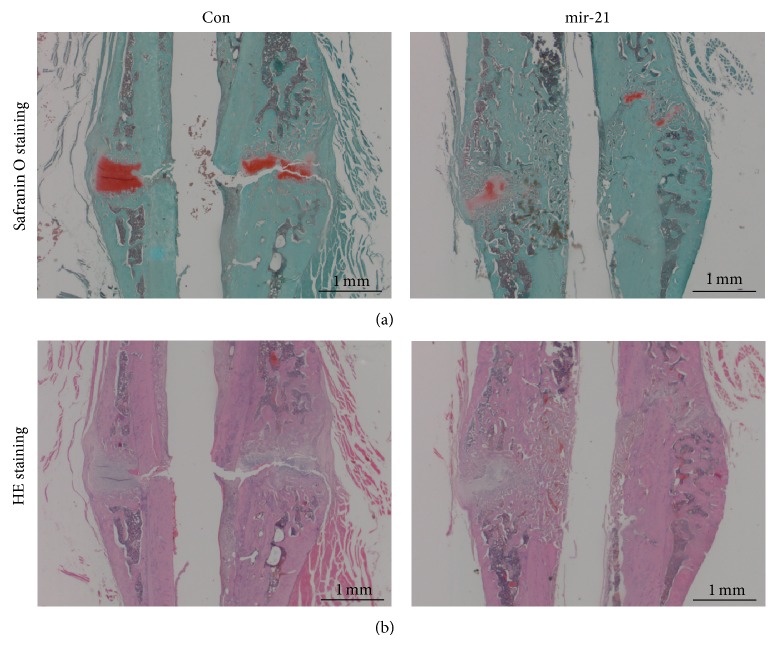
mir-21-MSCs intervention accelerated endochondral ossification via histological analysis. (a) Representative sections stained with Safranin O indicated endochondral ossification partially accomplished in mir-21-MSCs intervention group. In contrast, lots of uncalcified chondrocytes which remained in fracture site implied delayed healing. (b) The result of HE staining showed that the continuity of bone had been recovered and bone remodeling was taking place vigorously in the mir-21-MSCs intervention group, while cracks still could be detected and bone remodeling was inconspicuous in control group.

Representative sections from 2 groups stained with HE and Safranin O were shown in Figure 3. Much more chondrocytes could be detected in the control group than mir-21 group, which means that the newly formed chondrocytes have not been mineralized completely in control. In contrast, most of the callus had been calcified and the continuity of the cortical bone had been almost recovered.

### 3.4. Mechanical Testing

**Figure 4:**
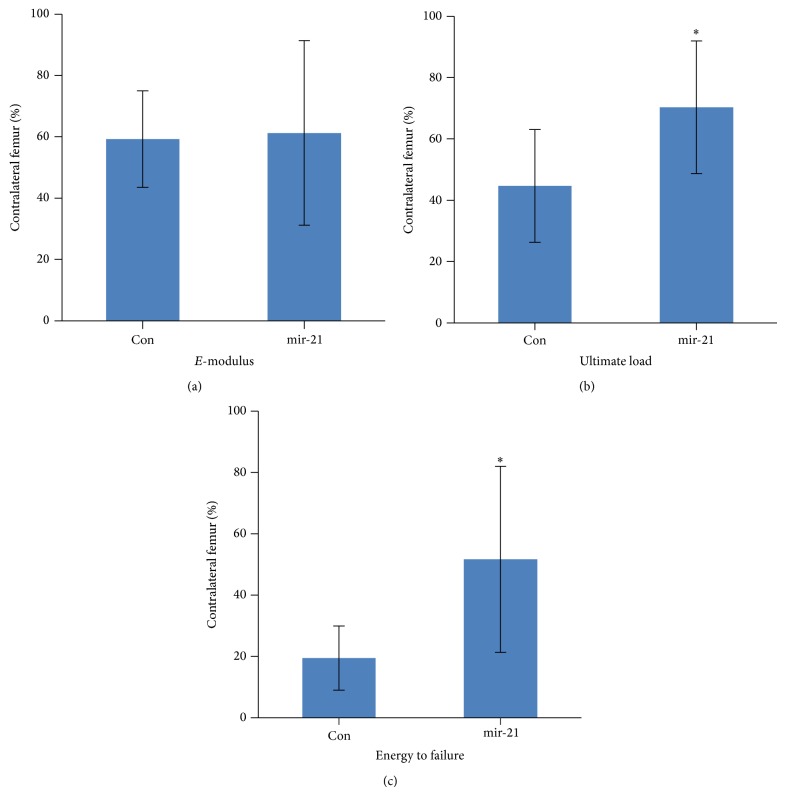
mir-21-MSCs intervention resulted in better mechanical properties of fractured femur. The mechanical properties (including ultimate load, energy to failure, and *E*-modulus) in the fractured femur were normalized with contralateral intact femur (in percent). ^*^ *P* < 0.05 compared with control, *n* = 8.

To investigate the ultimate outcome of healing quality after mir-21-MSCs intervention, mechanical testing was performed to detect the specimens' biomechanical properties. The results showed a significant improvement in ultimate load and the energy to failure in mir-21-MSCs group after being normalized with the contra-lateral intact femur (Figure 4). But there was no significant difference between these two groups in *E*-modulus, which is recognized as the tissue stiffness. The data before the normalization were shown in Table 1. The result indicated that fracture healing was better in toughness after mir-21-MSCs intervention, with the same stiffness recovery.

## 4. Discussion

MicroRNAs have been proven to play important roles in regulation of the complex process of osteogenic differentiation and osteoblastic bone formation. For a promising clinical application of miRNAs therapy, we investigated a specific miRNA, mir-21, which is signature for osteogenic regulation.

Numerous studies characterize mir-21 function in relation to its target(s) during skeletal disorders, such as osteoporosis [32], osteoarthritis [33], and osteosarcoma [34]. Considering that the expression change of miRNAs could reflect these kinds of disorders and the phenotype differentiation could be altered by some specific miRNAs expressed at high level in MSCs target tissue-specific regulators, maybe overexpression of mir-21 in MSCs could be the potential targets for clinical intervention. To our knowledge, it is the first time to study whether overexpression of mir-21 could enhance bone formation in fracture healing animal model, which is the necessary preparation for clinical application.

Previous study has demonstrated that mir-21 is upregulated during the process of osteogenesis differentiation in MSCs [23]. The results from the current study showed that overexpression of mir-21 could accelerate the formation of calcium nodule during osteogenesis differentiation, which is a functional marker of mineralization [35]. In order to confirm the effect of mir-21 on osteogenic differentiation of rBMSCs, we also detected the expression level of some osteogenesis-related gene markers, such as OPN, Runx2, Osterix, and ALP. OPN is a prominent bone matrix protein produced by osteoblastic cells [36]. Runx2 is a pivotal transcription regulator and plays crucial roles in osteoblast differentiation [37]. ALP is an early marker of osteoblastic differentiation [38]. ALP hydrolyzes pyrophosphate and generates inorganic phosphate to promote mineralization, suggesting that ALP plays an important role in bone formation [39, 40]. In this study, we found that overexpression of mir-21 significantly increased the expression levels of OPN and ALP at two different time points, which strongly suggested that mir-21 enhanced osteogenic differentiation of rBMSCs* in vitro*.

Furthermore, we retrieved the target gene of mir-21 with prediction software on website (Diana Lab, TargetScan, FindTar) and found that SOX-2 may be the target gene of mir-21, which is a negative regulator in Wnt signal pathway. Therefore, we detected the expression level of mir-21 and SOX-2 three days after osteogenic induction by q-PCR; the result proved our prediction that SOX-2 could be an effective target of mir-21. In addition, recent researches have confirmed our prediction that mir-21 can downregulate SOX-2 protein expression by binding to the 3′-UTR of it [41, 42], which may partly explain how mir-21 exerts its biological function* in vitro* and* in vivo*.

Next, we further investigated the effect of mir-21 on fracture healing by administrating mir-21 transfected rBMSCs in a closed femur fracture model of rats. According to the result of micro-CT, we found that mir-21 intervention could increase the new bone formation and mineralization. And this effect was confirmed by histological analysis result. Bone remodeling, which is recognized as the last stage of fracture healing, was found to take place vigorously in mir-21-MSCs treatment group. Conversely, most of the cartilage from the fracture site in control group still remained uncalcified in the callus. In addition, we quantified the healing quality of cicatrized bone by mechanical test and found that elevation of mir-21 in rBMSCs demonstrated a better result in mechanical properties, including ultimate load and energy to failure. Taken together, these results suggested that mir-21-MSCs intervention could significantly accelerate bone fracture healing in the model of SD rats* in vivo*.

Besides, the microenvironment* in vivo* is more complicated than that* in vitro*. Our unpublished data indicated that rBMSCs could still be observed in fracture site at 4 weeks after transplantation, implying that some rBMSCs could survive in the new environment and contribute to bone fracture healing. In this study we demonstrated the positive role of mir-21 in osteogenesis in fracture healing model, which was a paramount promise for clinical application of miRNAs therapy.

In conclusion, we have demonstrated that overexpression of mir-21 could improve osteogenesis in rBMSCs and accelerate rats fracture healing in a closed femur fracture model. As far as we know, most of the researches related to mir-21 in osteogenesis were carried out* in vitro*, while very little information was given about its regulation function* in vivo*, especially in fracture healing process. It is encouraging that our results which indicated elevation of mir-21 in rBMSCs showed a positive effect on fracture healing* in vivo*. Most importantly, this study expands our previous understanding on the osteogenic functions of mir-21, suggesting that it could be a potential therapeutic target for fracture healing.

## Supplementary Material

For regular qPCR, beta-actin was used as internal control. Tested genes include OPN, Runx2, Osx, and ALP. For mir-21 quantification, U6 was as internal control. The universal primer was supplied in the kit.

## Figures and Tables

**Table 1:** Mechanical test of the fractured femur (*n* = 8).

	*E*-modulus (Mpa)		Max force (N)		Energy between (J)	
	Normal	Fracture	Normal	Fracture	Normal	Fracture
Con	47.45 ± 3.86	28.47 ± 6.82	197.10 ± 19.73	85.97 ± 23.23	0.10 ± 0.02	0.03 ± 0.01
mir-21	46.74 ± 4.89	27.94 ± 12.53	170.61 ± 14.03	113.35 ± 32.53	0.07 ± 0.01	0.06 ± 0.02
