# Supplementary material for: mir-21 Overexpressing Mesenchymal Stem Cells Accelerate Fracture Healing in a Rat Closed Femur Fracture Model
Source: Biomed Res Int. 2015 Mar 23;2015:412327. doi: 10.1155/2015/412327 (PMC4386680; doi:10.1155/2015/412327)
Supplement: Supplementary file 1 — For regular qPCR, beta-actin was used as internal control. Tested genes include OPN, Runx2, Osx, and ALP. For mir-21 quantification, U6 was as internal control. The universal primer was supplied in the kit. [file 412327.f1.pdf]

Supplementary table 1. Sequences of primers for real time PCR.

Gene name	*Accession number*	*Forward primer (5' to 3')*	*Reverse primer (5'to 3')*
OPN	NM_012881.2	TCCAAGGAGTATAAGCAGCGGGCCA	CTCTTAGGGTCTAGGACTAGCTTCT
Runx2	NM_001278483.1	CCGATGGGACCGTGGTT	CAGCAGAGGCATTTCGTAGCT
Osx	NM_181374.2	GGAGGTTTCACTCCATTCCA	TAGAAGGAGCAGGGGACAGA
ALP	NM_013059.1	TCCGTGGGTCGGATTCCT	GCCGGCCCAAGAGAGAA
β-actin	NM_031144.3	CGTAAAGACCTCTATGCCAACA	CGGACTCATCGTACTCCTGCT
U6	NR_004394	CTCGCTTCGGCAGCACA	AACGCTTCACGAATTTGCGT
miR-21	NR_029493	GCCTAGCTTATCAGACTGATGTTGA	Universal primer
